# Perineuronal Nets Enhance the Excitability of Fast-Spiking Neurons

**DOI:** 10.1523/ENEURO.0112-16.2016

**Published:** 2016-07-27

**Authors:** Timothy S. Balmer

**Affiliations:** Grass Laboratory, Marine Biological Laboratory, Woods Hole, Massachusetts 02543

**Keywords:** auditory brainstem, extracellular matrix, fast spiking, inhibition, perineuronal net, white noise current

## Abstract

Perineuronal nets (PNNs) are specialized complexes of extracellular matrix molecules that surround the somata of fast-spiking neurons throughout the vertebrate brain. PNNs are particularly prevalent throughout the auditory brainstem, which transmits signals with high speed and precision. It is unknown whether PNNs contribute to the fast-spiking ability of the neurons they surround. Whole-cell recordings were made from medial nucleus of the trapezoid body (MNTB) principal neurons in acute brain slices from postnatal day 21 (P21) to P27 mice. PNNs were degraded by incubating slices in chondroitinase ABC (ChABC) and were compared to slices that were treated with a control enzyme (penicillinase). ChABC treatment did not affect the ability of MNTB neurons to fire at up to 1000 Hz when driven by current pulses. However, *f*–*I* (frequency–intensity) curves constructed by injecting Gaussian white noise currents superimposed on DC current steps showed that ChABC treatment reduced the gain of spike output. An increase in spike threshold may have contributed to this effect, which is consistent with the observation that spikes in ChABC-treated cells were delayed relative to control-treated cells. In addition, parvalbumin-expressing fast-spiking cortical neurons in >P70 slices that were treated with ChABC also had reduced excitability and gain. The development of PNNs around somata of fast-spiking neurons may be essential for fast and precise sensory transmission and synaptic inhibition in the brain.

## Visual Abstract

**Figure:**
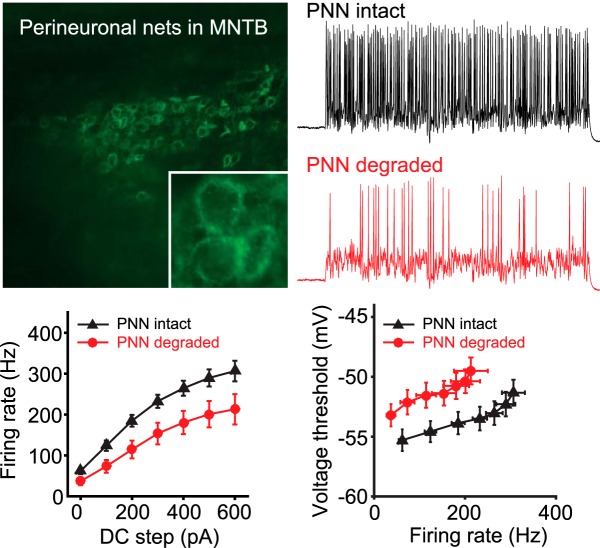

## Significance Statement

Perineuronal nets (PNNs) are extracellular matrix specializations that surround the somata of fast-spiking inhibitory neurons in most areas of the brain. Although PNN development correlates with the restriction of plasticity and their disruption causes enhancement of plasticity *in vivo*, it is unclear how PNNs affect the neurons they surround. In the present study, mature neurons were stimulated with fluctuating currents to measure their input/output functions after degradation of PNNs with the enzyme chondroitinase. Both the medial nucleus of the trapezoid body principal neurons and parvalbumin-expressing fast-spiking cortical interneurons treated with chondroitinase exhibited reduced excitability compared with control-treated cells. Increased spike threshold may underlie this change in gain. Thus, PNNs increase the evoked activity of fast-spiking neurons and could control plasticity by enhancing synaptic inhibition.

## Introduction

Perineuronal nets (PNNs) are specialized complexes of extracellular matrix molecules that form around the somata of neurons throughout the brain (Brückner et al., 1993; Bertolotto et al., 1996; Celio et al., 1998). PNNs are made up of an array of proteoglycans and polysaccharides that give the cell surface a strong negative charge (Seeger et al., 1994; Morawski et al., 2004, 2015). In particular, PNNs tend to surround fast-spiking neurons (Blosa et al., 2013; Sonntag et al., 2015). Whether this negatively charged PNN coat contributes to fast-spiking activity is unclear.

Current understanding of how PNNs contribute to brain processes comes largely from studies in which PNNs are degraded enzymatically. Disruption of PNNs reactivates plasticity in the adult visual cortex (Pizzorusso et al., 2002, 2006; Carulli et al., 2010; Vorobyov et al., 2013), promotes collateral sprouting in the denervated brainstem (Massey et al., 2006), and allows the recovery of spinal cord function after injury (Galtrey et al., 2007; García-Alías et al., 2009). There are several hypothesized mechanisms through which the degradation of PNNs may control plasticity in mature brains. PNNs may prevent structural remodeling of dendrites (Mataga et al., 2004; McGee et al., 2005; Fawcett, 2009; Shen et al., 2009; Giger et al., 2010) or neurotransmitter receptor mobility and plasticity (Bukalo et al., 2001; Frischknecht et al., 2009; Kochlamazashvili et al., 2010). Given the important role that PNNs play in plasticity, surprisingly little is known about what PNNs contribute to the physiology of the neurons that they surround. It is possible that the disruption of PNNs *in vivo* caused a change in neuronal excitability, in addition to known structural and molecular changes that occur.

PNNs surround fast and precisely spiking neurons throughout the vertebrate brain (Härtig et al., 1992, 1999; Murakami et al., 1994; Balmer et al., 2009). PNNs are common in the auditory brainstem (Bertolotto et al., 1996; Härtig et al., 2001; Blosa et al., 2013), which contains some of the fastest and most precisely firing neurons. The auditory brainstem circuit transmits signals from the periphery with precision and speed in order to compare the timing and loudness of sounds between the two ears (Trussell, 1997, 1999; Oertel, 1999; Ashida and Carr, 2011). Differences in spike timing are used to compute the location of a sound in the environment of the animal. Principal neurons in the medial nucleus of the trapezoid body (MNTB) can follow extremely fast afferent stimulation (>1000 Hz) with incredible accuracy (Kim et al., 2013). The development of reliable fast spiking in MNTB occurs after postnatal day 14 (P14; Taschenberger and von Gersdorff, 2000), which correlates with the formation of PNNs around the principal neurons (Myers et al., 2012). The increase in spike reliability has been attributed to changes to the anatomy of the large axosomatic calyx of Held synapse, which is the main input to MNTB principal neurons (Taschenberger et al., 2002). PNN development may provide additional optimization for fast-spiking activity.

The role of PNNs in the physiology of fast-spiking neurons was tested by recording from mature mouse MNTB principal neurons and parvalbumin-expressing cortical interneurons in acute brain slices. Chondroitinase-treated cells had a marked decrease in evoked activity and a consistent delay relative to control-treated cells. The development of PNNs around somata of fast-spiking neurons may tune fast sensory transmission and inhibition in the brain.

## Materials and Methods

### Slice preparation

C57BL/6 mice of both sexes between P21 and P28 were used for MNTB recordings. CB6-Tg(Gad1-EGFP)G42Zjh/J (RRID:IMSR_JAX:007677; Chattopadhyaya et al., 2004) mice of both sexes >P70 were used for cortical neuron recordings. Mice were anesthetized with isoflurane and decapitated following the standards of humane care developed by the National Institutes of Health and the Society for Neuroscience, and procedures were approved by the Marine Biological Laboratory Institutional Animal Care and Use Committee. The brain was rapidly extracted into ice-cold high-sucrose artificial cerebrospinal fluid (ACSF) containing the following (in mm): 230 sucrose, 25 glucose, 2.5 KCl, 3 MgCl_2_, 0.1 CaCl_2_, 1.25 NaH_2_PO_4_, 25 NaHCO_3_, 0.4 ascorbic acid, 3 myo-inositol, and 2 Na-pyruvate, pH 7.4, saturated with 95% O_2_ and 5% CO_2_ (Huang and Trussell, 2011). Coronal sections 200 μm thick for MNTB and 300–400 μm thick for cortex were prepared using a vibratome (VT1200S, Leica). Immediately after cutting, slices were incubated in 35°C recording ACSF containing either chondroitinase ABC (ChABC) or penicillinase (P-ase) for 1 h, followed by storage at room temperature. Recording ACSF contained the following (in mm): 125 NaCl, 25 glucose, 2.5 KCl, 1 MgCl_2_, 2 CaCl_2_, 1.25 NaH_2_PO_4_, 25 NaHCO_3_, 0.4 ascorbic acid, 3 myo-inositol, 2 Na-pyruvate, and ∼305 mOsm, pH 7.4, saturated with 95% O_2_ and 5% CO_2_ (Huang and Trussell, 2011).

### Enzymatic degradation of PNNs

Immediately after slicing, brain slices were incubated in 0.2 U/ml ChABC (catalog #2905, Sigma-Aldrich) or control enzyme P-ase (catalog #P0389, Sigma-Aldrich) in recording ACSF for 1 h at 35°C in a small slice chamber (BSK2, Scientific Systems Design). ChABC is a well characterized enzyme that degrades PNNs by removing glycosaminoglycan (GAG) side chains from chondroitin sulfate proteoglycans, reduces PNN labeling in acute slices (Bukalo et al., 2001), and reduces cell surface charge (Morawski et al., 2015).

### Whole-cell current-clamp recordings

Slices were transferred to a submerged recording chamber and superfused with ACSF heated to 33–35°C at 3–4 ml/min. Slices were viewed using infrared differential interference contrast (IR-DIC) and a 63× water-immersion objective (AxioExaminer, Zeiss) and camera (Flash4-LT, Hamamatsu). Pipettes were pulled from thick-walled borosilicate glass capillaries (1.5 mm outside diameter; WPI) to a tip resistance of 2–4 MΩ. The internal pipette solution contained the following (in mm): 113 K-gluconate, 4.8 MgCl_2_, 4 ATP, 0.5 Tris-GTP, 14 Tris-phosphocreatine, 0.1 EGTA, and 10 HEPES, pH 7.25 with KOH, ∼290 mOsm. Reported voltages are corrected for a −10 mV liquid junction potential. Whole-cell recordings were amplified and low-pass filtered (6 kHz; Multiclamp 700B, Molecular Devices) and digitized using pClamp software (50–100 kHz; Digidata 1550, Molecular Devices). MNTB neurons were verified by their physiological properties (low input resistance, transient firing pattern, and outward rectification) and in some cases were filled with a fluorophore (20 μm Alexa Fluor 594; catalog #A10438, Life Technologies) and recovered *post hoc*. Parvalbumin-expressing (PV+) cortical neurons were identified by EGFP expression and fast-spiking activity during positive current steps. Bias current was not used to maintain resting membrane potential in any cells.

### White noise current stimulation

The Gaussian noise current stimulus was synthesized in Matlab (MathWorks) by passing Gaussian white noise through an exponential filter with a correlation time of 0.5 ms (2 kHz; Bryant and Segundo, 1976; Slee et al., 2005; Mease et al., 2013). This 500 ms frozen noise stimulus was multiplied to create four noise levels [measured as the SD of the noise (0, 200, 400, and 600 pA SD) and superimposed on seven current steps (0–600 pA)]. Each cell was stimulated with all 28 levels of noise and a DC step five times in a pseudorandom order with 750 ms interstimulus intervals.

### Immunohistochemistry and microscopy

After recording, slices were fixed for 2 h overnight in 4% paraformaldehyde in 0.1 m phosphate buffer and rinsed in 0.1 m PBS. Slices were incubated in biotinylated *Wisteria floribunda* agglutinin (WFA; 20 μg/ml; catalog #L1516, Sigma-Aldrich) for 1 h. After rinsing in PBS, slices were incubated in streptavidin-Alexa Fluor 488 (20 μg/ml; catalog #S11223, Life Technologies; RRID:AB_2336881) for 1 h. Slices were mounted to microscope slides and coverslipped with 90% glycerol and 10% PBS, pH 9.0. Slices were imaged with an epifluorescence microscope (Imager.Z2 with Colibri LED System, Zeiss). For quantification of PNN brightness a 20×/0.8 numerical aperture objective (Plan Apochromat, Zeiss) was used, and consistent LED brightness (25%) and exposure time (150 ms) were maintained across all sections imaged. All immunohistochemistry was performed in parallel with the same reagents, and imaging was performed blinded to the treatment condition in the same imaging session. PNN labeling was quantified by measuring pixel intensities within an outlined area circumscribing MNTB (ImageJ; RRID:SCR_003070; Schneider et al., 2012).

### Data analysis

Clampfit (pClamp, Molecular Devices) was used to measure spike shape properties. Matlab was used for the analysis of pulse trains, voltage threshold, and white noise stimulation. Statistical analyses were performed in Matlab, Sigmaplot (SyStat), and Excel (Microsoft).

#### Spike shape analysis

Depolarizations that crossed −30 mV and had the shape of an action potential were considered to be spikes. Spike amplitudes were measured as the peak membrane potential relative to the preceding resting membrane potential. Spike widths were measured at half the spike amplitude. Rising slopes were based on 30–90% peak measurements, and falling slopes were based on 10–90% peak measurements. Input resistance was calculated as the slope of the linear portion of the current–voltage (*I–V*) curve (−150 to 0 pA, 500 ms steps). Inward rectification was estimated as the difference between the transient hyperpolarization and the steady-state membrane potential during a −500 pA step. Outward rectification was estimated as the difference between the steady-state response during a 450 pA step and an extrapolation of the linear portion of the *I–V* curve to 450 pA. This measure indicates the difference between the measured membrane potential during a 450 pA step and the predicted membrane potential, given a linear increase in membrane potential. Afterhyperpolarization (AHP) was measured as the difference between the resting membrane potential preceding the spike and the minimum membrane potential after the spike (or train of spikes).

#### Spike threshold

Spike voltage thresholds were calculated using a phase-plane analysis, plotting the membrane potential versus the dV/dt and measuring the voltage where dV/dt crossed an empirically defined level of 40 mV/ms. Other methods including 30–100 mV/ms, 2–3 SDs above the mean, and the peak of the first or second time derivative of voltage were also used, and the results were similar.

#### Relative spike delay

To quantify the difference between spike times in ChABC-treated cells vs P-ase-treated cells, a peristimulus time histogram (PSTH) was made by combining the first trial of each cell. Calculations were triggered every time at least 25% of the P-ase-treated cells spiked within a 0.25 ms bin. For each of these “spike events,” the mean time of spike peaks that occurred in the ChABC or P-ase-treated group within 1 ms were compared. A positive delay represents P-ase spikes leading the ChABC spikes.

## Results

### ChABC treatment effectively degraded PNNs in acute brain slices

**Figure 1.**
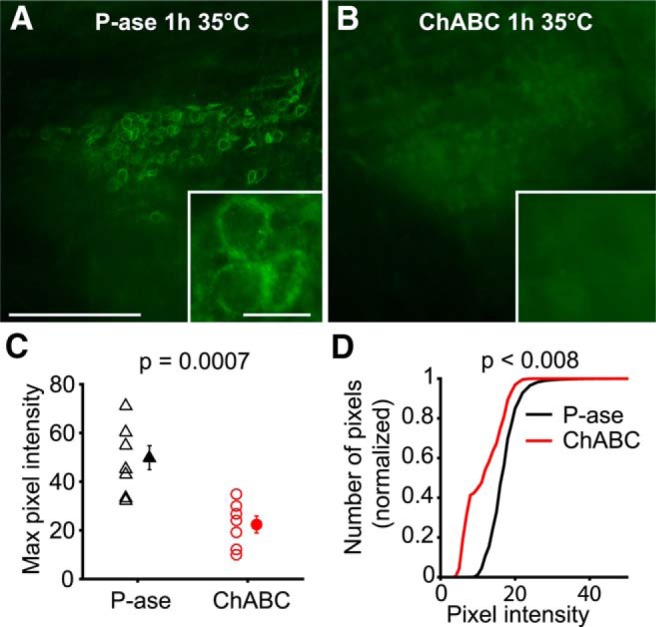
ChABC effectively degrades PNNs in acute brain slices during slice recovery. ***A***, WFA**-**labeled PNNs are visible surrounding MNTB neurons after P-ase treatment (negative control). Scale bars: 200 μm; inset, 20 μm. ***B***, ChABC-treated slices had reduced PNN labeling. ***C***, Maximum pixel intensities in MNTB were reduced by ChABC (*t* test, *p* = 0.0007). Error bars are the mean and SEM. ***D***, Cumulative plot of pixel intensities shows that ChABC-treated MNTB regions have reduced brightness (two-sample Kolmogorov–Smirnov, *p* < 0.008). Slices were labeled in parallel with the same reagents and imaged with the same microscope settings.

To test how PNNs affect the physiology of the cells that they surround, PNNs were either degraded or left intact in acute mouse brain slices. Slices were incubated in either ChABC, an enzyme that digests the GAG chains of the PNN, or P-ase as a negative control treatment. The slices were maintained in these solutions for 1 h at 35°C with constant 95% O_2_ and 5% CO_2_ saturation. In P-ase-treated control slices, there were many PNNs labeled in MNTB by the lectin WFA, which labels GAG chains (Fig. 1*A*). PNN labeling was greatly reduced by the 1 h ChABC treatment, although it was not completely abolished (Fig. 1*B*). The maximum pixel intensities within MNTB were significantly reduced by ChABC treatment (*t* test, *p* = 0.0007, *N* = 15 slices; Fig. 1*C*). The distribution of pixel intensities within MNTB was negatively shifted by ChABC treatment (two-sample Kolmogorov–Smirnov test, *p* < 0.008, *N* = 15 slices; Fig. 1*D*).

### PNN degradation did not affect passive properties, spike shape, or spike failures

**Figure 2.**
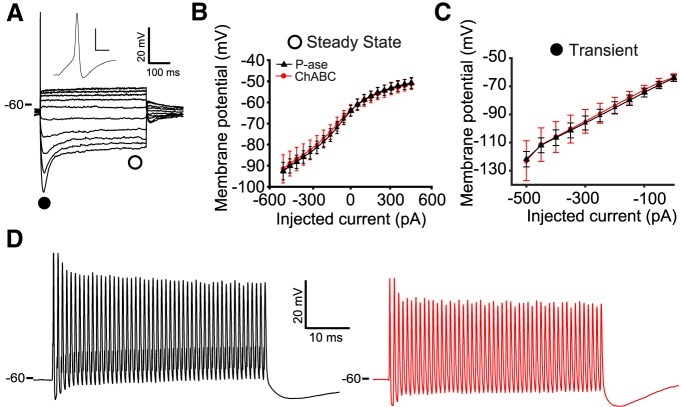
Passive membrane properties were not affected by ChABC treatment. ***A***, Response to current steps show that hyperpolarization activated transient, outward rectification and a single spike on depolarizing steps. Inset shows a spike with an extended time base. Inset calibration: 20 mV, 1 ms. ***B***, ChABC treatment did not affect the *I–V* curve measured when the membrane reached steady state (as indicated by the open circle in ***A***), or ***C***, size of the hyperpolarizing transient, measured at the peak (filled circle in ***A***). Error bars are the mean and SEM. ***D***, Both P-ase-treated (left) and ChABC-treated (right) neurons were able to fire at 1000 Hz in response to a train of 4 nA, 0.1 ms pulses.

Whole-cell current clamp recordings were made from MNTB-containing slices that were treated with ChABC or P-ase. Slices treated with either enzyme were indistinguishable in appearance from untreated slices under IR-DIC imaging. The presence of PNNs did not affect the ability to make low-resistance whole-cell recordings. There was no effect of treatment condition on resting membrane potential, input resistance, or inward or outward rectification (*t* tests, *p* > 0.05; Fig. 2*A–C*; Table 1), suggesting that ChABC treatment did not alter the viability of the neurons. Moreover, any differences observed in spiking activity are unlikely to be caused by changes in passive membrane properties.

**Figure 3.**
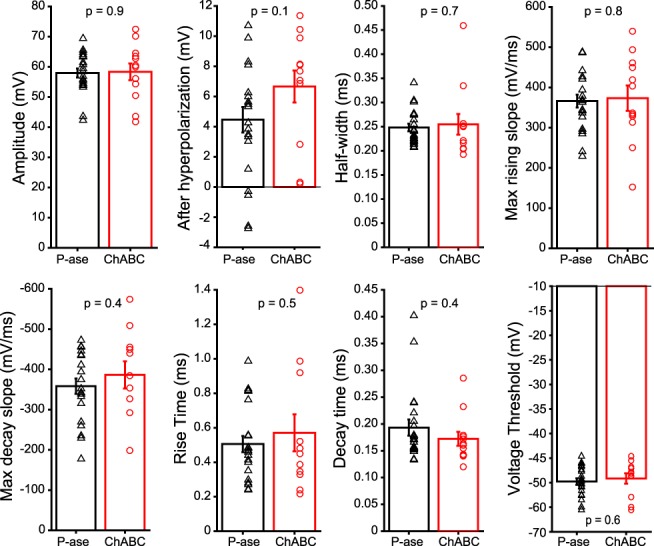
Spikes evoked by 450 pA, 500 ms current step were not affected by ChABC treatment. Data points are individual cells. Error bars are the mean and SEM.

To investigate the effect of PNNs on spike shape, a single action potential was evoked by a 450 pA, 500 ms step or a 4 nA, 0.1 ms pulse, which lead to different spike shapes (Johnston et al., 2009). Properties of action potential shape and timing were not significantly different between ChABC- and P-ase-treated groups for either protocol (*t* tests, *p* > 0.05; Table 2; Fig. 3). Trains of action potentials were elicited by a series of 50 4 nA, 0.1 ms pulses at rates of 100, 300, 500, 800, and 1000 Hz. All cells spiked in response to every current pulse at 100, 300, and 500 Hz. Some cells failed at 800 and 1000 Hz, but there were no statistically significant differences between the treatment groups (χ^2^ tests, *p* > 0.05). Example traces for 1000 Hz stimulation are shown in Figure 2*D*. Spike properties including the measurements that were used for single spikes in Figure 3 were quantified across the 50 spikes in the train. None of these properties were significantly affected by ChABC treatment at any of the tested frequencies. The action potential amplitude tended to be 5–10 mV shorter in the ChABC-treated cells, although this difference did not reach statistical significance. In addition, the afterhyperpolarization at the end of the train (of 50 successful spikes) and the interspike potential were not different between treatment groups at any stimulation frequency (*t* tests, *p* > 0.05). Thus, PNNs were not necessary for MNTB neurons to spike at up to 1000 Hz in response to large square currents.

### ChABC treatment reduced the excitability of MNTB neurons

**Figure 4.**
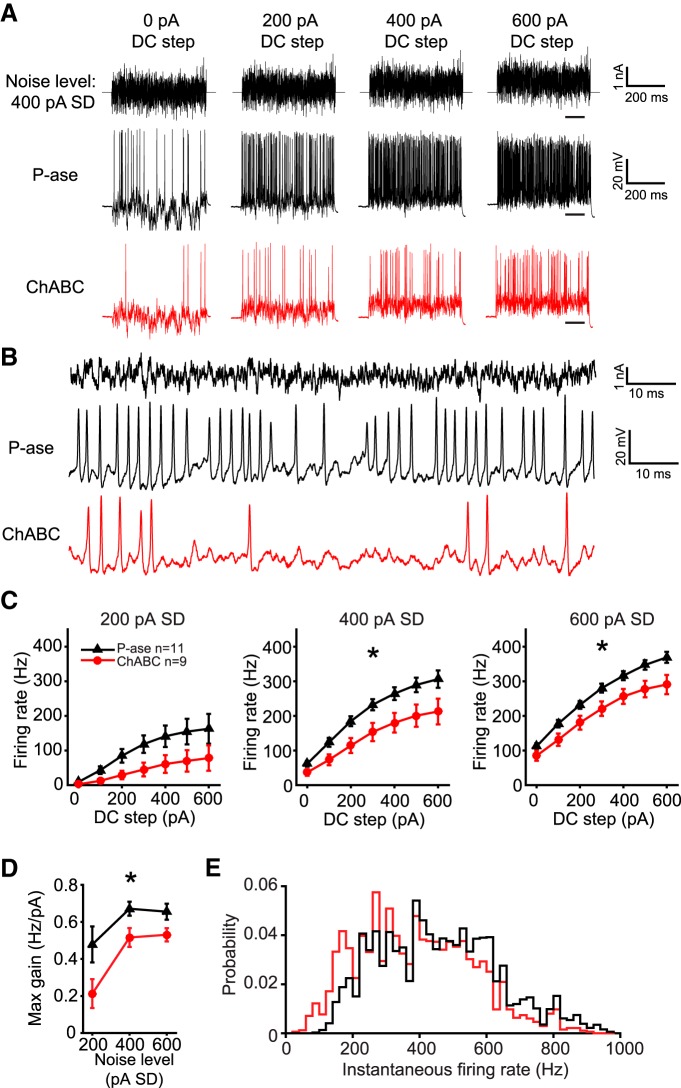
ChABC-treated MNTB neurons exhibited lower gain. ***A***, Example white noise currents with noise level of 400 pA SD at four levels of DC step (top), spiking response in a P-ase-treated cell (middle, black), and spiking response in a ChABC-treated cell (bottom, red). ***B***, Expanded time base showing spiking responses from the traces in ***A*** at the time indicated by underlining in ***A*** in the 600 pA DC step condition. ***C***, *f*–*I* curves indicate significantly lower firing rates in ChABC-treated cells compared with P-ase-treated cells (*two-way RM ANOVA, *p* < 0.05). ***D***, Maximum gain calculated from the *f*–*I* curves was significantly lower in ChABC-treated cells, indicating reduced excitability (*two-way RM ANOVA, *p* < 0.05). ***E***, The distribution of instantaneous firing rates was shifted to lower frequencies and did not indicate a change in spiking pattern.

To investigate whether PNNs have an effect on the spike output of MNTB neurons in response to fluctuating currents, cells were stimulated with currents that contained a range of amplitudes and frequencies (Bryant and Segundo, 1976; Slee et al., 2005; Street and Manis, 2007; Mease et al., 2013). A Gaussian distributed “white noise current” was synthesized and filtered such that it would contain frequencies up to 2 kHz (see Materials and Methods). The same current was injected with varying gain (noise level), measured as the SD of the current (0-600 pA SD) and varying positive bias current (0-600 pA DC steps). Figure 4*A* shows an example white noise current with 400 pA noise level superimposed on four of the seven DC steps, and with an expanded time base in Figure 4*B*. All cells were stimulated with the same synthesized currents.

ChABC-treated cells fired markedly less in response to white noise current stimulation. Over all trials and levels of the white noise currents, the ChABC-treated cells fired on average 67.6% the number of spikes that P-ase-treated cells fired (mean ± SD; P-ase cells: 10,031 ± 2775, *n* = 11; ChABC: 6783 ± 3108, *n* = 9; *t* test, *p* = 0.024). Figure 4*A* shows an example trace from a P-ase-treated (black) and a ChABC-treated neuron (red) at the 400 pA SD noise level superimposed on four DC steps. This approach produced high-quality frequency–intensity (*f*–*I*) curves for these cells, which are not possible with DC steps alone due to their transient firing behavior (Fig. 4*C*). ChABC-treated cells spiked significantly less at noise levels of 400 and 600 pA compared with P-ase-treated cells [two-way repeated-measures (RM) ANOVAs, interaction *p* < 0.05; Fig. 4*C*]. The maximum slope of the *f*–*I* curves, which indicates the gain of firing as the DC steps increase, was lower in ChABC-treated cells at all noise levels (two-way RM ANOVA, *p* = 0.03; Fig. 4*D*). The distribution of the instantaneous spike rates was shifted to lower frequencies in ChABC-treated cells, but the overall shape of the distribution was similar to P-ase-treated cells (Fig. 4*E*). This suggests that the change in firing rate was not due to a change in spiking pattern. For example, if the ChABC-treated cells started firing in bursts with large interburst intervals, the distribution would show an increase in high instantaneous firing rates and a decrease in low rates. In sum, ChABC-treated cells were less excitable than P-ase-treated cells.

**Figure 5.**
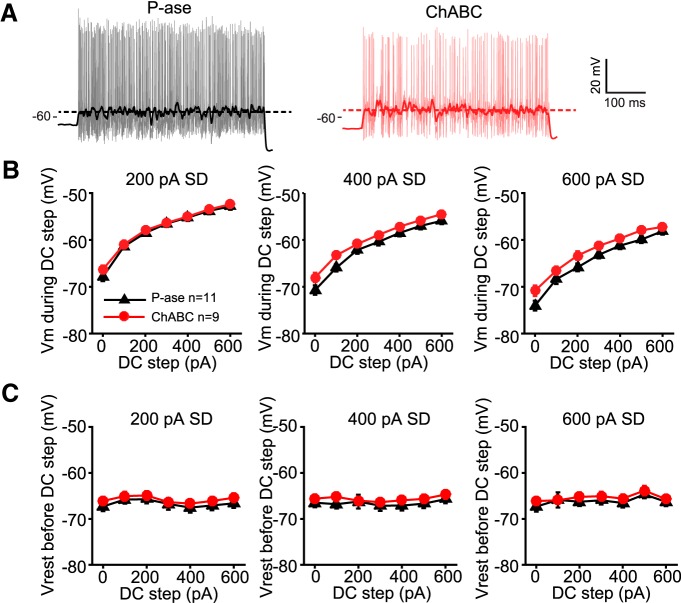
Input resistance and resting membrane potential were not different between treatment groups. ***A***, Traces were low-pass filtered to calculate the membrane potential during white noise current stimulation. Thick dark line is the filtered trace plotted over the original trace. Dashed horizontal line indicates the mean membrane potential (Vm) during the current step. These values were used to compare the membrane potential during DC steps shown in ***B***. Examples traces are at the 400 pA noise level and 600 pA DC step. ***B***, There were no significant differences between treatment groups at any noise level (two-way RM ANOVA, *p* > 0.05). ***C***, Resting membrane potential (Vrest) was calculated as the membrane potential before the current injection. There were no differences between treatment groups at any noise level (two-way RM ANOVA, *p* > 0.05).

Importantly, these differences in firing rate were not due to differences in membrane potential depolarization during the steps, which was measured by filtering out the spikes (two-way RM ANOVA, *p* > 0.05; Fig. 5*A*,*B*). This agrees with the finding that the input resistance of ChABC-treated cells was not different than P-ase-treated cells (Table 1). The resting membrane potential measured between the steps was also not different between groups (two-way RM ANOVA, *p* > 0.05; Fig. 5*C*). Thus, the reduced excitability of MNTB neurons treated with ChABC is not due to changes in passive membrane properties.

**Table 1:** MNTB neuron passive properties were not different between treatment groups

Passive properties of MNTB neurons			
	Penicillinase (*n* = 15)	Chondroitinase (*n* = 10)	*p* values (unpaired *t* tests)
Resting membrane potential (mV)	−64 ± 2.1	−63 ± 1.8	0.405
Input resistance (MΩ)	76 ± 31.5	72 ± 22.9	0.365
Inward rectification (mV)	−24 ± 9.5	−20 ± 7.0	0.354
Outward rectification (mV)	−32 ± 7.9	−34 ± 16.6	0.661

Values are reported as the mean ± SD, unless otherwise indicated.

### Spike-triggered average shows ChABC-treated cells required more current to spike

**Figure 6.**
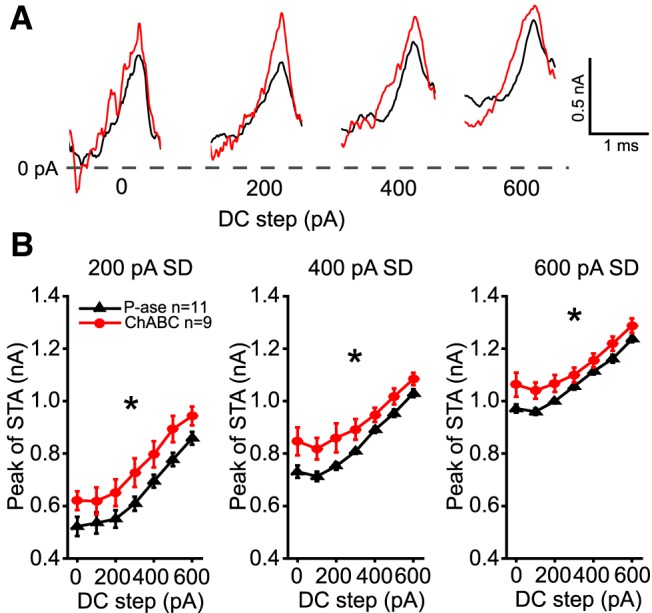
STAs of injected currents reveal higher current threshold in ChABC-treated MNTB neurons. Injected currents that triggered spikes were aligned and averaged. ***A***, Overlaid examples of P-ase-treated (black) and ChABC-treated (red) cells at the 400 pA SD noise level superimposed on four DC current steps. The peaks of the action potentials were aligned with the rightmost point of each STA. Note that in these examples the peak of the STA is higher in the ChABC-treated cell than in the P-ase-treated cell. ***B***, The average STA peak across cells was significantly higher among the ChABC-treated cells than the P-ase-treated cells (*two-way RM ANOVA, *p* < 0.05). Error bars are the mean and SEM.

The currents that were injected immediately prior to each spike were averaged within each level of noise and DC step. Figure 6*A* shows example spike-triggered averages (STAs) for a cell treated with P-ase (black) and for another cell treated with ChABC (red) across four DC steps. The peak of the STAs was significantly higher during the 200, 400, and 600 pA noise levels in the ChABC-treated cells (two-way RM ANOVAs, *p* < 0.05; Fig. 6*B*). This indicates that the ChABC-treated cells required larger currents to spike. The slopes of the STA currents were not different between groups, nor were their preceding troughs.

### Spike threshold was more depolarized in ChABC-treated cells

**Figure 7.**
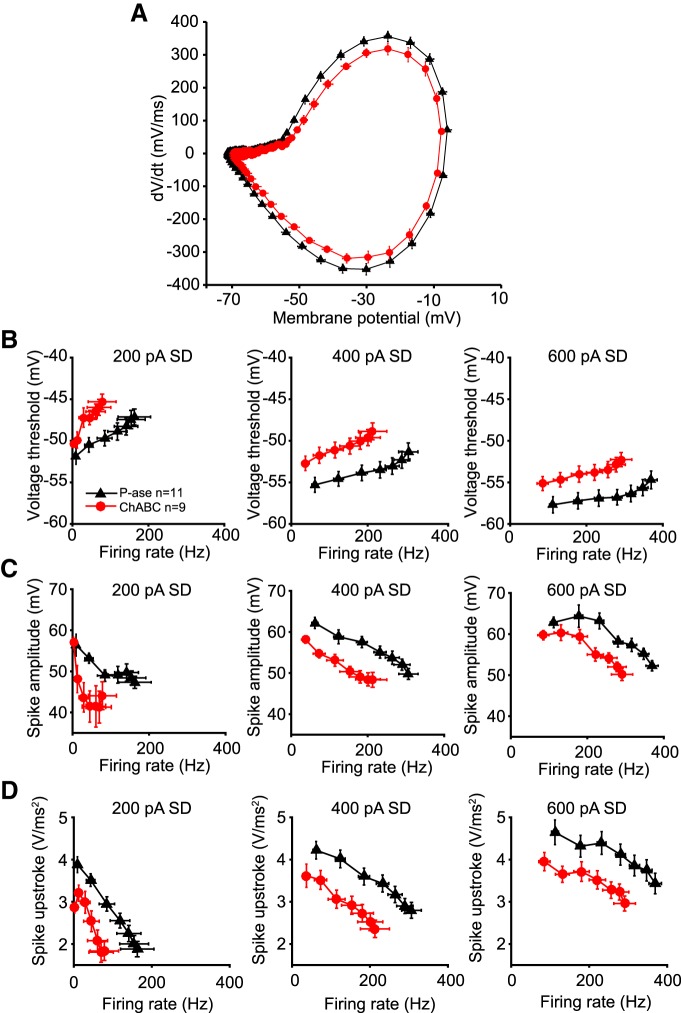
Voltage threshold and spike shape during fast spiking. ***A***, Phase plane plots of white noise evoked spikes during 400 pA SD noise level without a DC step averaged across ChABC-treated (red) and P-ase-treated (black) cells. The membrane potential where the dV/dt begins to increase is more depolarized in ChABC-treated cells, indicating a more depolarized voltage threshold. Also note that the upstroke of the spike is slower (lower peak dV/dt) and reaches a lower membrane potential. ***B***, Voltage thresholds were significantly depolarized in ChABC-treated cells compared with P-ase-treated cells (see Results). Each marker indicates a DC step (from 0 to 600 pA) plotted at the average evoked firing rate and the average voltage threshold during the step. ***C***, Spike amplitude was significantly smaller in ChABC-treated cells. ***D***, The acceleration of the membrane potential was significantly lower in ChABC-treated cells. Error bars are the mean and SEM.

ChABC treatment did not affect passive properties that might explain the observed reduction in spiking, such as input resistance or resting membrane potential (Table 1, Fig. 5). A change in the voltage threshold for spikes could underlie the change in spike rate. Spike thresholds were calculated by making a phase-plane plot of the membrane potential and measuring the voltage at which the dV/dt crossed an empirically defined level of 40 mV/ms (Fig. 7*A*; see Materials and Methods). The variance of the voltage thresholds during each noise current injection was high, but did not differ between treatment groups. Because the firing rate can affect voltage thresholds (Henze and Buzsáki, 2001), comparisons between treatment groups were made across DC steps that evoked comparable firing rates. Voltage thresholds of ChABC-treated cells were significantly depolarized at firing rates of 0–50, 50–100, and 100–150 Hz during the 400 pA SD noise level, and at 150–200 Hz during the 600 pA noise level, compared with P-ase-treated cells (*t* tests, *p* < 0.05; Fig. 7*B*).

Spike amplitude of ChABC-treated cells were also reduced at firing rates of 0–50 Hz during the 200 pA SD noise level, 50–100 and 100–150 Hz during the 400 pA noise level, and 150–200 Hz during the 600 pA SD noise level, compared with P-ase-treated cells (*t* tests, *p* < 0.05; Fig. 7*C*). The speed of the upstroke of the spikes evoked by white noise stimulation was slower in the ChABC-treated cells (Fig. 7*D*). The acceleration of the membrane potential was significantly slower in ChABC-treated cells at firing rates of 50–100 Hz during the 400 pA SD noise level, and 150–200 and 200–250 Hz during the 600 pA noise level (*t* tests, *p* < 0.05). These observations are consistent with a change in a Na^+^ conductance after PNN degradation.

### Spikes in ChABC-treated cells had a delayed onset

**Figure 8.**
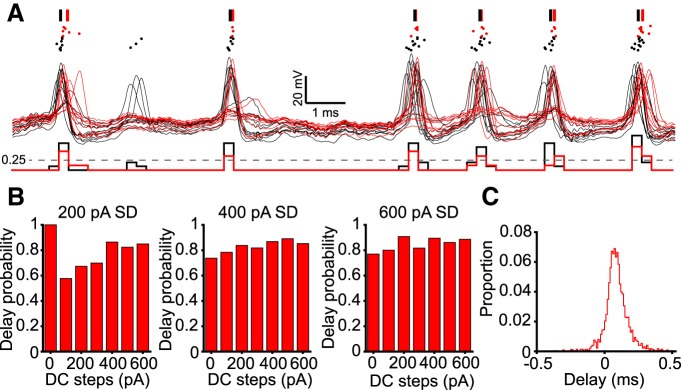
ChABC-treated cells consistently fired later than P-ase-treated cells. ***A***, Each overlaid trace is from a different P-ase-treated (black) or ChABC-treated (red) cell. A dot raster is plotted above the spike trains, with each row indicating a different cell. The PSTH below indicates how spike events were identified. When a 0.25 ms bin reached a threshold where ≥25% of P-ase-treated cells fired, a mean spike time was calculated for spikes occurring within 1 ms centered on the bin. The vertical lines above indicate the mean spike time of the spikes within each 1 ms spike event. ***B***, The probability of the ChABC-treated cells to fire later than the P-ase-treated cells during these spike events was high (> 50%) across all levels of gain and DC steps. ***C***, The distribution of the delays of spike events is shifted to the right, indicating that spikes usually occurred later in ChABC-treated cells than P-ase-treated cells.

If spike threshold was depolarized in ChABC-treated cells and passive membrane properties were not affected, then they should fire later in response to the identical sample of white noise current, compared with P-ase-treated cells. Indeed, when spike trains evoked by the same white noise current were overlaid, it became apparent that the ChABC-treated cells not only did not fire each time the control cells did, but when they fired the spikes occurred later. Figure 8*A* shows overlaid traces of every P-ase-treated (11 cells, black) and ChABC-treaded cell (9 cells, red). The mean spike times of the spikes that occurred within a 1 ms time window are indicated as vertical lines above the traces. Overall, 86% of these spike events were later in the ChABC-treated group than the P-ase-treated group, with an average relative delay of ∼70 μs (Fig. 8*B*,*C*). There was no difference in the jitter of the spikes between groups.

### Cortical inhibitory interneurons were similarly affected by ChABC

To test the generality of these results, cortical fast-spiking interneurons of >P70 mice were recorded. Older mice were used for these recordings than MNTB, because PNNs develop much later in the cortex than in the brainstem (Pizzorusso et al., 2002). GFP-expressing cells in the CB6-Tg(Gad1-EGFP)G42Zjh/J mouse line are presumed to be PV+ inhibitory interneurons (Chattopadhyaya et al., 2004).

**Figure 9.**
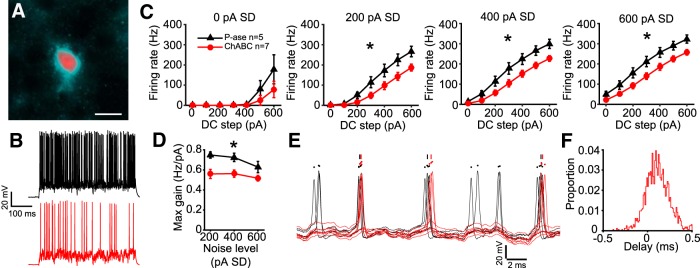
Parvalbumin-expressing fast-spiking cortical neurons were similarly affected by ChABC. ***A***, Example of a PV+ GFP**-**expressing cell that was filled with a fluorophore during recording (Alexa Fluor 594; red) and labeled *post hoc* with WFA (cyan). Scale bar, 10 um. ***B***, Example traces showing responses to white noise current (400 pA SD, 300 pA DC step) in P-ase-treated cells (black) and ChABC-treated cells (red). ***C***, Fast-spiking cortical neurons treated with ChABC spiked significantly less than P-ase-treated cells in response to white noise currents (*two-way RM ANOVA, *p* < 0.05). ***D***, Maximum gain calculated from the *f*–*I* curves was significantly lower in ChABC-treated cells, indicating reduced excitability (*two-way RM ANOVA, *p* < 0.05). ***E***, Overlaid traces from each P-ase-treated (black) and ChABC-treated (red) cell, illustrating that ChABC-treated cells fired spikes later than P-ase-treated cells. ***F***, Histogram showing rightward shift in delay, indicating that most spike events from ChABC-treated cells were delayed relative to P-ase-treated cells.

GFP-expressing cells in layers 4–6 of somatosensory cortical slices were patched, and their fast-spiking phenotype was verified by injecting positive DC current steps without noise. In some cases, GFP^+^ cells were filled with a fluorophore and labeled with WFA to verify that they had PNNs in the P-ase-treated group, and had attenuated PNNs in the ChABC-treated group (Fig. 9*A*). The resting membrane potential was not different between treatment groups (mean ± SD; P-ase: −76 ± 4.5 mV, *n* = 5; ChABC: −80 ± 5.7 mV, *n* = 7; *t* test, *p* = 0.257), nor was input resistance (P-ase: 82 ± 33.1 MΩ, *n* = 5; ChABC 73 ± 20.3 MΩ, *n* = 7; *t* test, *p* = 0.558).

The same white noise current that was used for MNTB neurons (Fig. 4) was injected into the fast-spiking cortical neurons. The ChABC-treated PV+ neurons fired significantly less in response to the white noise currents than P-ase-treated cells (two-way RM ANOVAs, *p* < 0.05; Fig. 9*B*,*C*). ChABC-treated cells fired 64.1% of the number of spikes that P-ase-treated cells fired in response to the white noise currents (mean ± SD; P-ase-treated: 9074 ± 2681, *n* = 7; ChABC-treated: 5813 ± 1845, *n* = 5; *t* test, *p* = 0.031). These neurons were less sensitive to the noise level than MNTB neurons, firing at similar rates at 200, 400, and 600 pA noise SD levels. The gain of spiking was lower in ChABC-treated cells compared with P-ase-treated cells (two-way RM ANOVA, *p* = 0.03; Fig. 9*D*). The spikes that occurred in ChABC-treated cells were delayed relative to those in P-ase-treated cells (Fig. 9*E*). A total of 65.5% of the spike events of ChABC-treated cells occurred after those of the P-ase-treated cells during the white noise current stimulation (Fig. 9*F*). Thus, both cortical PV+ fast-spiking interneurons and MNTB principal neurons were affected in a similar way by PNN degradation. In both cases, the excitability of the cells was reduced. PNNs may be important for fast-spiking neurons in general to tune responses to input.

## Discussion

PNNs have been correlated with the maturation of fast-spiking neurons, but their role in spiking behavior has remained unclear (Härtig et al., 1999). In this study, enzymatic degradation of PNNs reduced spiking in two types of fast-spiking inhibitory neurons. MNTB principal neurons receive a single large synaptic input, are glycinergic, and project out of MNTB. Cortical PV+ neurons receive many synaptic inputs to their dendrites, are GABAergic, and are interneurons. PNN degradation had no observable effect on passive membrane properties, but reduced and delayed spiking in both cell types. The reason that ChABC-treated MNTB neurons fired less was not due to an inability to fire at high rates, because current pulses were able to drive spikes without failures. Instead, ChABC-treated cells had lower gain, firing less to fluctuating currents than control-treated cells. Thus, PNNs may enhance the gain of spike output in response to synaptic input during fast-spiking activity. Because PNNs typically surround inhibitory neurons, the development of PNNs may be a mechanism that enhances synaptic inhibition throughout the brain.

These results are consistent with a study that recorded extracellular spiking activity of MNTB principal cells *in vivo* in brevican knock-out mice, which have attenuated PNNs (Blosa et al., 2015). MNTB neurons in brevican knock-out mice fired at lower frequencies in response to sound than wild-type mice, and the sound pressure level threshold for evoking spikes was increased (Blosa et al., 2015). This change in spiking could not be attributed to an increase in transmission failures at the calyx of Held (Blosa et al., 2015). The present study suggests that the decrease in sound-evoked spiking in the brevican knock-out mouse may be due to reduced excitability of the MNTB principal neurons.

In a recent study investigating the role of PNNs in synaptic plasticity, ChABC treatment did not affect the *f*–*I* curves of CA2 hippocampal pyramidal neurons (Carstens et al., 2016). In contrast to the cells studied here, ChABC treatment significantly reduced the input resistance of these CA2 neurons (Carstens et al., 2016). This may have prevented the current pulses from causing the same level of membrane depolarization in the ChABC-treated cells and might explain why these neurons did not fire less after PNN degradation. Alternatively, PNNs may play different roles in neurons that do not fire at high frequencies. Whole-cell recordings have been made in dissociated cultures, which develop PNN-like structures (Miyata et al., 2005; Dityatev et al., 2007). Dissociated hippocampal interneuron cultures that were treated with ChABC had a reduced current threshold and reduced AHP compared with those of controls (Dityatev et al., 2007). These effects were not observed in this study, which is not suprising given that the PNNs likely develop differently in culture.

This is the first study to characterize frequency/intensity input/output functions of MNTB principal neurons. MNTB principal neurons fire a single spike in response to depolarizing current steps, which prevents the construction of meaningful *f*–*I* curves. White noise stimulation was an effective and sensitive approach to reveal the excitabilty of these neurons. Moreover, in mice and other mammals with high-frequency hearing, MNTB neurons are unlikely to phase lock to physiologically relevant high-frequency sounds (Kopp-Scheinpflug et al., 2008). A fluctuating current that contains frequencies that are within the range of the hearing sensitivity of a mouse (∼1–100 kHz) may be more similar to *in vivo* synaptic currents than traditional stimulation protocols consisting of current pulses delivered at lower frequencies (<1 kHz).

This study focused on the effect of PNNs on nonsynaptic physiology because PNNs generally surround the soma and, in some cases, the axon initial segment and proximal dendrite. Other extracellular matrix molecules surround synapses, but generally these components are less dense than the PNN around the soma. The calyx of Held is a notable exception and is an attractive synapse to study the effect of PNNs, because PNN components are both inside and outside the cleft (Blosa et al., 2013). This study refuted the hypothesis that PNNs are necessary for fast spiking, at least in response to trains of 4 nA current pulses. These current pulses were smaller than the initial excitatory postsynaptic current at the calyx of Held during a train, but were larger than currents at the end of a train, which are reduced by short-term synaptic depression. Spontaneous activity that occurs *in vivo* may reduce the synaptic currents further (Hermann et al., 2007). It is possible that PNNs reduce transmission failures due to depressed synaptic currents during ongoing physiological activity.

ChABC injection into the brain *in vivo* has been reported to enhance plasticity in adulthood in the visual cortex, perirhinal cortex, and amgdala (Pizzorusso et al., 2002, 2006; Gogolla et al., 2009; Romberg et al., 2013), but the mechanism by which this occurs remains quite unclear. The results reported here may shed light on how ChABC could enhance plasticity. For example, ocular dominance plasticity is reactivated by ChABC injections into the visual cortex (Pizzorusso et al., 2002, 2006) and by genetic manipulations of PNNs (Carulli et al., 2010). A variety of treatments that alter the excitatory/inhibitory balance of the visual cortex has been reported to control ocular dominance plasticity (Takesian and Hensch, 2013). Specific pharmacogenetic inhibition of PV+ neurons in visual cortex extends the critical period for ocular dominace plasticity (Kuhlman et al., 2013). Reducing PV+ neuron activity may affect ocular dominance plasticity by shifting the ability for synapses to undergo synaptic plasticity (Kirkwood and Bear, 1994; Harauzov et al., 2010; Kuhlman et al., 2010). The present study suggests that ChABC injections into the visual cortex may reduce PV+ cell spiking *in vivo* and thereby allow the synaptic plasticity that shapes binocular visual responses.

How might PNN degradation affect neuronal excitability? PNNs may prevent the diffusion of ion channels along the plasma membrane. AMPA receptors have been shown to diffuse more after the degradation of PNN-like structures that form around cultured hippocampal neurons (Frischknecht et al., 2009). Perhaps voltage-dependent Na^+^ and K^+^ channels that underlie spiking are maintained in clusters or prevented from being endocytosed by interactions with PNNs. Indeed, the voltage-dependent Na^+^ Channel Na_V_1.2 interacts with tenascin-R (Srinivasan et al., 1998), a major component of the PNN, although the function of this interaction has not been investigated.

It is possible that the voltage dependence of ion channels is affected by the PNN. PNNs are highly negatively charged (Morawski et al., 2015). Removal of this negative charge at the membrane could affect the local electric field sensed by the gating subunits of ion channels. This could have an effect that is similar to the extracellular application of high divalent cation solutions, which neutralize the membrane surface charge, shift channel gating to more depolarized membrane potentials, and reduce spiking (Frankenhaeuser and Hodgkin, 1957; Hille, 2001). The removal of PNNs could thus shift the activation of voltage-dependent ionic currents to higher voltages. In this case, PNNs may increase excitability by increasing the negative charge of the membrane and shift channel gating of one or more channel species to more hyperpolarized potentials.

Another way that neuronal excitability could be affected by ChABC is by reducing electrostatic interactions between the PNNs and cations. PNNs have been proposed to be a buffering system to maintain a stable microenvironment of Na^+^ and K^+^ ions (Härtig et al., 1999). Although not further investigated here, this proposed buffering system may explain why ChABC treatment affected spike shape during fast spiking (evoked by the white noise currents; Fig 7), but not spikes evoked by current pulses (Table 2; Fig 3). Whether PNNs attract these Na^+^ and K^+^ ions is unclear. However, ChABC increases the diffusion of calcium ions in cortical and hippocampal brain slices, but not the monovalent cation tetramethylammonium (Hrabetová et al., 2009). Increased diffusion of calcium ions could have an effect similar to the application of extracellular high divalent solutions. Future work is necessary to test these hypotheses.

**Table 2:** MNTB neurons spike shape properties were not different between treatment groups

Spike shape properties of MNTB neurons						
	450 pA current step			4 nA, 0.1 ms current pulse		
	Penicillinase (*n* = 21)	Chondroitinase (*n* = 12)	*p* values (unpaired *t* tests)	Penicillinase (*n* = 6)	Chondroitinase (*n* = 7)	*p* values (unpaired *t* tests)
Amplitude (mV)	58 ± 6.9	58 ± 9.6	0.914	59 ± 3.7	56 ± 6.7	0.398
AHP (mV)	4.8 ± 2.28	6.7 ± 3.73	0.125	7.3 ± 2.08	6.6 ± 3.62	0.658
Half-width (μs)	248 ± 35.8	255 ± 74.3	0.750	185 ± 24.3	194 ± 29.2	0.530
Maximum rising slope (mV/ms)	366 ± 73.1	373 ± 109.1	0.820	696 ± 62.0	629 ± 170.5	0.384
Maximum decay slope (mV/ms)	−358 ± 87.4	−386 ± 116.9	0.436	−399 ± 44.6	−371 ± 45.5	0.296
Rise time (μs)	132 ± 33.7	160 ± 126.5	0.348	64 ± 8.8	72 ± 30.7	0.534
Rise slope (mV/ms)	291 ± 82.5	291 ± 117.6	0.987	591 ± 97.9	542 ± 162.3	0.535
Decay time (μs)	193 ± 68.9	172 ± 45.3	0.340	159 ± 22.9	164 ± 16.4	0.668
Decay slope (mV/ms)	−273 ± 85.3	−304 ± 104.1	0.352	−311 ± 41.7	−286 ± 31.5	0.227
Latency from current onset (ms)	1.02 ± 0.276	1.11 ± 0.473	0.475	0.28 ± 0.017	0.31 ± 0.093	0.340

Values are reported as the mean ± SD, unless otherwise indicated.

PNNs are complex structures and are likely to have diverse roles in neuronal physiology and plasticity (Tsien, 2013). The present study shows that PNNs increase the gain of the inhibitory neurons they surround and could therefore increase synaptic inhibition in the brain. In the auditory system, neurons must fire rapidly, reliably, and precisely in order to process small differences in timing between the two ears. It is not surprising then, that so many neurons in the auditory brainstem are coated with PNNs. It remains to be seen whether the disruption of PNNs that can occur in disease underlies auditory pathology.

## Synthesis

Reviewing Editor: Leonard Maler, University of Ottawa Decisions are customarily a result of the Reviewing Editor and the peer reviewers coming together and discussing their recommendations until a consensus is reached. When revisions are invited, a fact-based synthesis statement explaining their decision and outlining what is needed to prepare a revision will be listed below. The following reviewer(s) agreed to reveal their identity: Ethan Goldberg.

Editorial comments

The reviewers are both now much more enthusiastic about this manuscript. The first reviewer finds that, although control experiments would be very useful, they are not absolutely essential. The author no longer has access to the transgenic mice and the Ms does provide an advance in an important and perhaps neglected field. On this basis, the first reviewer does agree that this Ms is acceptable for publication in eNeuro.

The second reviewer emphasizes our lack of understanding of the role of the extracellular matrix in neuronal excitability. This reviewer gives specific suggestions concerning additional analyses and changes in the figures that would improve this Ms. Most of these can be done without additional experiments. Below, I have indicated (bold text) the changes that are most important as well as those not absolutely essential.

I recommend that the author make the necessary revisions and carefully explain how the Ms has been changed.

This Ms will not require re-review but I will require full justification of the revisions made before reaching a final Editorial decision.

Comments of both reviewers are presented, in full, below.

Reviewer #1

The key finding of this manuscript is that enzymatic degradation of the extracellular matrix structure termed the perineuronal net (PNN) surrounding two types of neurons in the brain that fire at high frequency -- principal neurons of the medical nucleus of the trapezoid body, and parvalbumin-positive fast-spiking GABAergic neocortical interneurons -- changes the excitability of these cells in response to noisy current injections simulating naturalistic synaptic inputs, but does not alter high-frequency firing. Hence, the manuscript provides an advance in the field in that it describes a cellular neurophysiological consequence of perineuronal net degradation in these two cell types that prominently display PNNs. I must admit that this result remains somewhat puzzling to me, and the author does not further explore the mechanism(s) by which the main finding occurs. Such additional experiments may be beyond the scope of this work. There is no question that there are control experiments missing that would strengthen the manuscript. I am not sure if such experiments are “essential” per se, but these could in principle be done without unreasonable additional effort. However, the author states that such experiments cannot be done due to no longer having access to the necessary transgenic mice. The author did include further information in the Discussion section that I found to be thoughtful and performed some additional data analysis to investigate the issues raised in the previous review.

The author has provided a different presentation of the data (graph rather than table) in the rebuttal that I find very convincing and could be included in a final version of the manuscript.

Editor: Addition of this graph in the final Ms would be very useful.

I must admit that I remain somewhat puzzled regarding the basic finding of this paper; however, I think that the experiments and analysis are well done and that further in-depth investigation of the mechanism whereby the findings obtain is perhaps beyond the scope of this work. I do think that there are control experiments missing that would strengthen the manuscript. The author has stated that such experiments cannot be done due to no longer having access to the necessary transgenic mice. The author did include further information in the Discussion section that I found to be thoughtful, and performed some additional data analysis to investigate the issues raised in the previous review. I think that this paper will be a nice addition to the literature on the functional role of the perineuronal net.

Reviewer #2

The extracellular matrix structure (ECM) plays important roles in axonal nodal formation as well as in synaptic plasticity in the CNS. Understanding the role of extracellular matrix structure such as PNN in determining the excitability of fast spiking neurons will provide more information about its specific functions to regulate intrinsic properties of fast spiking neurons. However the author did not address the cellular mechanisms of the main finding in this manuscripts. Some data need to be carefully analyzed.

Some figures are not representative. For example, in Figure 2D, representative traces of action potential trains from P-ase treated group and ChABC-treated group look different. ChABC-treated group shows a strong adaption of spikes and larger AHP, and also more hyperpolarization after the train (>−10 mV). Author should consider carefully whether this change is significant.

Editor: Please carefully address this criticism and, if necessary, include more representative traces.

The author states that a goal of the work is to investigate the role of PNNs in fast-spiking neurons (MNTB and PV+ neocortical interneurons). The author suggests that degradation of the PNN reduced excitability in those neurons, and increased spike threshold “likely” underlies this change. There is no further investigation as to the underlying mechanisms of increased spike threshold, although previous reviewers suggested further studies in sodium channel expression and function after ChABC treatment.

1. The author should carefully analyze the spike shape properties the consistent series resistance during whole-cell recordings. If the series resistance increased during recordings, it would affect the spike shape of MNTB neurons.

Editor: Please analyze the spike shapes and address this comment according to the results of this analysis.

In Table 1 and 2, author address the resting membrane potentials are -64 mV (P-ase) and −63mV (ChABC) and the amplitude of spikes are 58 mV and 58 mV. It is assumed that the author measured the amplitude of spike from the resting membrane potential to the peak of spike. The peak of spike did not reach 0 mV. (No overshooting?) In whole-cell recording of MNTB principal neurons, step-current injection (450 pA) generated action potential with the amplitude of > 80 mV. It would be better to include the representative trace of a single spike evoked by 450-pA current step from MNTB neurons (or Expanded figure of Figure 2A to the inset), and indicate how to measure the amplitude and half width of action potentials and AHP.

Editor: Add the representative trace; this might be added as an insert to an exisiting figure.

The author recorded the AHP with two different protocols: one is 450 pA current injection and the other is the train (4 nA, 0.1ms, 50 pulses). It would be better to indicate how AHP was measured in each representative traces.

Editor: Use a blow up of an AHP to indicate how the AHP was measured; this can go into an insert within the appropriate figure.

2. The threshold of action potential could be reliably measured by searching the inflection point of spikes using step current injections. Thus, it is possible to measure voltage or current threshold for action potential generation via somatic current injection for MNTB neurons. Although the author states that the main finding is an alteration in spike threshold in PNN degraded neurons, the author did not address the threshold of spike using the step-like square current injection.

Editor: This is not an essential criticism. Typically, an extremum in the first or second derivative is used to find the inflection point and this is used as an estimate of the action potential voltage threshold. If at all possible, the author should do this analysis. Otherwise, the author should clearly state why such an analysis is not possible.

3. Changes in Na channel expression pattern (the length of AIS and location) impact on neuronal excitability and action potential firing. Voltage-dependent Na and K channels of MNTB neurons may be affected by PNN degradation, which is worth testing. In the rebuttal letter, the author wrote “I would respectfully argue that some of these changes could affect the spike threshold without necessarily affecting passive or active membrane properties”. After investigating thoroughly and addressing that passive or active membrane properties do not affect the spike threshold, then the author would like to explain the other possibility. This revision still lacks experiments to examine the involvement of voltage-dependent Na channels in changes in the spike threshold. Without immunostaining of Na and K channel expressions of the AIS of MNTB neurons, and no recordings of voltage-dependent Na and K currents, the author cannot rule out the changes in active membrane properties in these neurons after ChABC treatment. These immunostaining and electrophysiological recordings are feasible experiments in MNTB neurons.

Editor: Not an essential criticism. The immunostaining experiments are, in my opinion, beyond the scope of this study.

4. The author states, “I'm not sure whether a ChABC-induced change in the spike threshold of MNTB neurons could be seen with single spikes evoked by somatic current pulses or steps.” Even if the author is not sure, he or she did not test it. Without further supporting evidences that passive or active membrane properties are not associated with this increased threshold of spikes and reduced excitability in PPN degraded MNTB or PV+ neocortical interneurons, the author should consider toning down the statement: “increased spike threshold”.

Editor: Yes agree, please tone down this statement.

5. For Figure 1, it needs to address the morphological and structural analysis of MNTB neurons after the application of ChABC and penicillinase, and compared them with normal MNTB neurons (without any pre-treatment). For example, immunolabeling MNTB principal neurons with MAP2 or NeuN will confirm that ChABR specifically degrade PNNs without any structural alterations of MNTB neurons.

Editor: Not an essential criticism. The immunostaining experiments are, in my opinion, beyond the scope of this study.
